# Robot-assisted ipsilateral partial nephrectomy with distal ureterectomy for synchronous renal and ureteric tumors—a case report

**DOI:** 10.1186/s43046-022-00151-2

**Published:** 2022-11-28

**Authors:** Anandan Murugesan

**Affiliations:** Department of Urology, Kovai Medical Center and Hospital, Post Box No. 3209, Avinashi Road, Coimbatore, Tamilnadu, IN 641014 India

**Keywords:** Robotic partial nephrectomy, Renal cancer, Transitional cell cancer, Robotic distal, Ureterectomy, Renal dysfunction

## Abstract

**Background:**

Ipsilateral synchronous renal and ureteric tumor is uncommon. Nephron sparing surgery is the standard for small renal masses. Ureteric tumors can be selectively managed with nephron sparing surgery, especially in renal dysfunction. This case report details the management of double malignancy by nephron sparing surgery with robot-assisted approach.

**Case report:**

A 63-year-old gentleman with diabetes presented with history of intermittent gross hematuria for 2 weeks. He was clinically normal. On evaluation, he had grade 4 renal dysfunction (Se. creatinine 4.5 mg%) with mild proteinuria. Magnetic resonance imaging revealed right renal upper polar Bosniak III lesion and right hydroureteronephrosis due to 2 cm ureteric tumor near the vessel crossing. Renogram showed overall GFR of 22 ml/min with 31% (6 ml/min) contribution from the right side. He underwent robot-assisted right partial nephrectomy with distal ureterectomy and Boari flap ureteric reimplantation. Histopathology revealed margins free T2 clear cell carcinoma (kidney) and high-grade T3 transitional cell carcinoma (ureter). His nadir creatinine at 1 year follow-up was 3.3 mg% and no recurrence on MRI, cystoscopy, and ureteroscopy at 1 year.

**Conclusion:**

Minimally invasive nephron sparing surgery is feasible and reasonable option with satisfactory oncological control even in ipsilateral synchronous renal and ureteric tumors in selected patients with renal dysfunction.

## Background

Ipsilateral, synchronous renal parenchymal, and ureteric tumors are rare [1]. Nephron sparing surgery is the standard for small renal tumors [2]. In ureteric transitional cell carcinoma, nephron sparing surgery is being offered, in those with renal dysfunction [3]. Minimally invasive approach is preferred if technical expertise is available, especially for nephron sparing surgery [4]. This case report describes single stage management of ipsilateral synchronous renal parenchymal and ureteric tumors with robot-assisted partial nephrectomy and distal ureterectomy.

## Case presentation

A 63-year-old gentleman, on treatment for diabetes mellitus, presented with complaints of intermittent, total, painless, and hematuria for 2 weeks. He had no other systemic symptoms. His clinical examination was unremarkable. Biochemical evaluation showed serum creatinine of 4.5 mg%. Urinalysis showed mild proteinuria and glycosuria, along with hematuria. Ultrasonogram revealed right sided moderate hydronephrosis with upper polar complex cyst.

**Fig. 1:**
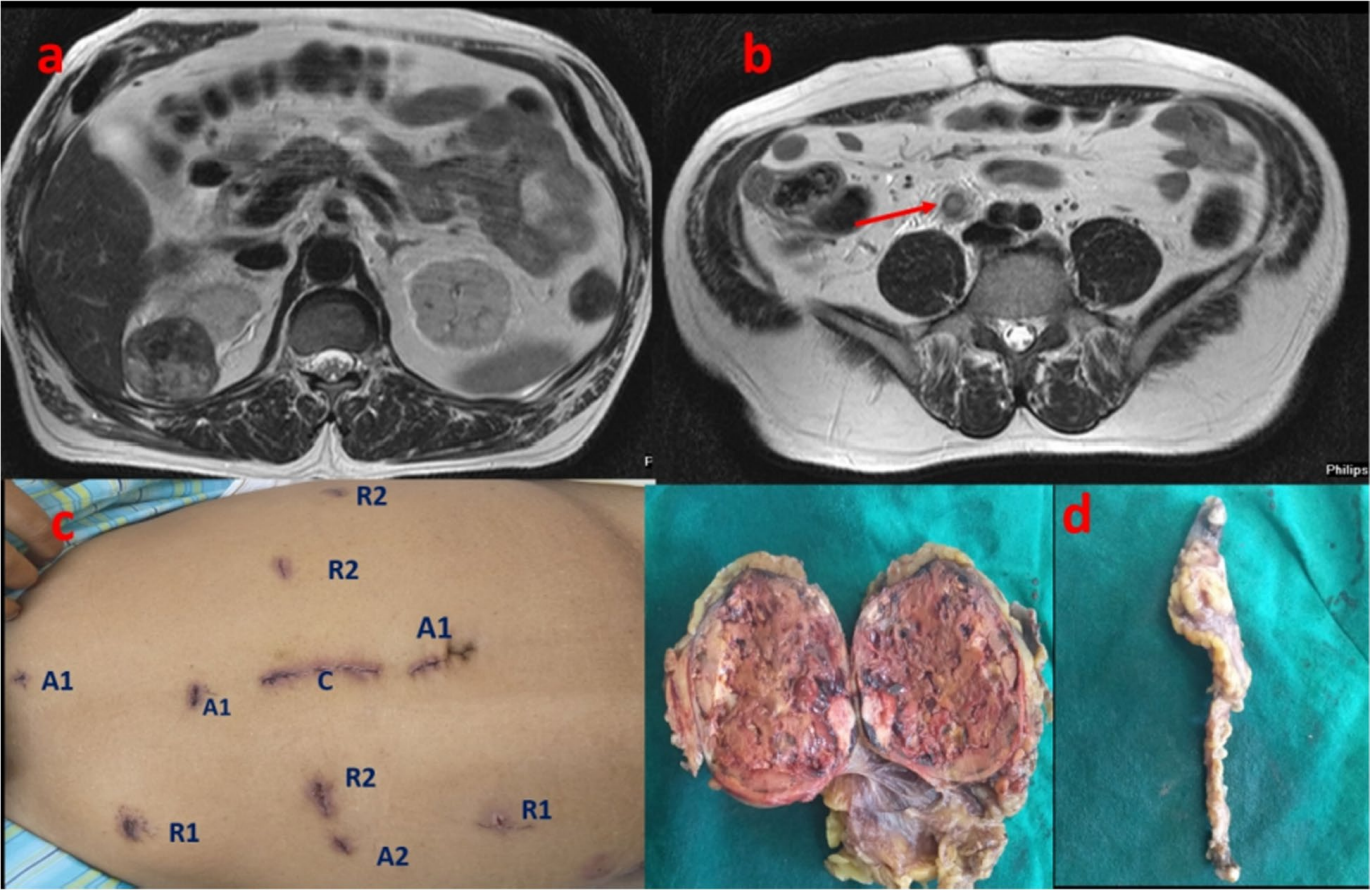
**a** MRI image of renal tumor. **b** MRI image of ureteric tumor. **c** Port sites (1—nephrectomy, 2—ureterectomy). **d** Resected specimen

Abdominal magnetic resonance imaging findings included right upper polar Bosniak III cyst (T2W hyperintense cyst with hypointense contents, minimal diffusion restriction) and moderate hydroureteronephrosis due to 2 cm hyperintense mass lesion in the ureter at vessel crossing (Fig. 1). No significant lymphadenopathy. Ethyl cysteine renogram revealed GFR of 6 ml/min (31%) for the right kidney and 16 ml/min (69%) for the left kidney.

**Fig. 2:**
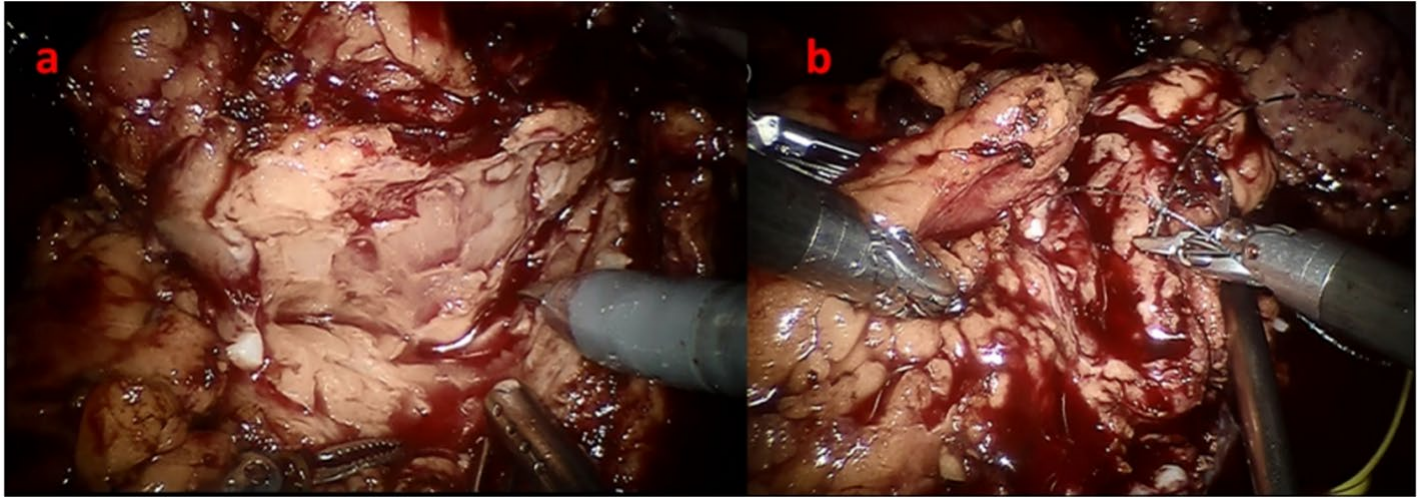
**a** Renal tumor being resected. **b** Renorraphy in progress

The gentleman was administered standard oral endotracheal anesthesia and cystoscopy was done to rule out bladder tumor. He was placed in left lateral position with kidney bridge elevation. Standard six ports of robotic partial nephrectomy (2 × 12 mm, 3 × 8 mm, 1 × 10 mm) were placed after creating pneumoperitoneum. Robot was docked from the side. Colon deflection, duodenum kockerization, hilar dissection, and vessel looping steps were performed as per standard description. Ureter was dissected and clipped proximal to the site of tumor based on visual cues and radiological correlation. Renal artery was clamped, tumor resected, and renorraphy done by sliding clip technique. Warm ischemia time (vein sparing) was 24 min. Ureter was divided proximal to the tumor and margin was sent for frozen section. Specimen bagged and robot was undocked (Fig. 2).

**Fig. 3:**
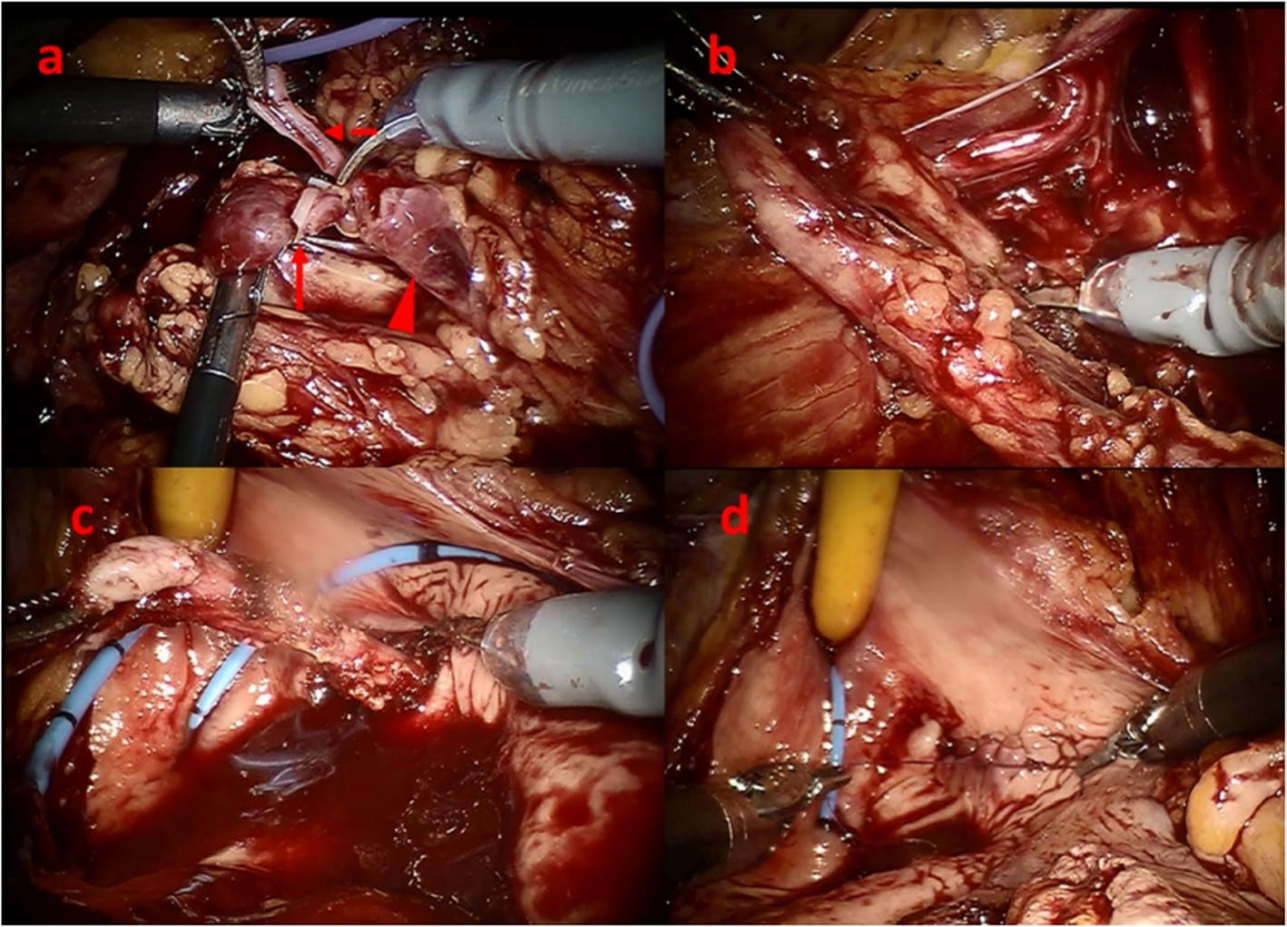
**a** Ureter clipped and divided, margin excised for biopsy (arrow—clipped ureter end, interrupted arrow—ureteric margin for biopsy, arrow head—proximal ureter). **b** Extra vesical dissection of ureter. **c** Intravesical dissection of ureter. **d** Ureteric orifice closure

**Fig. 4:**
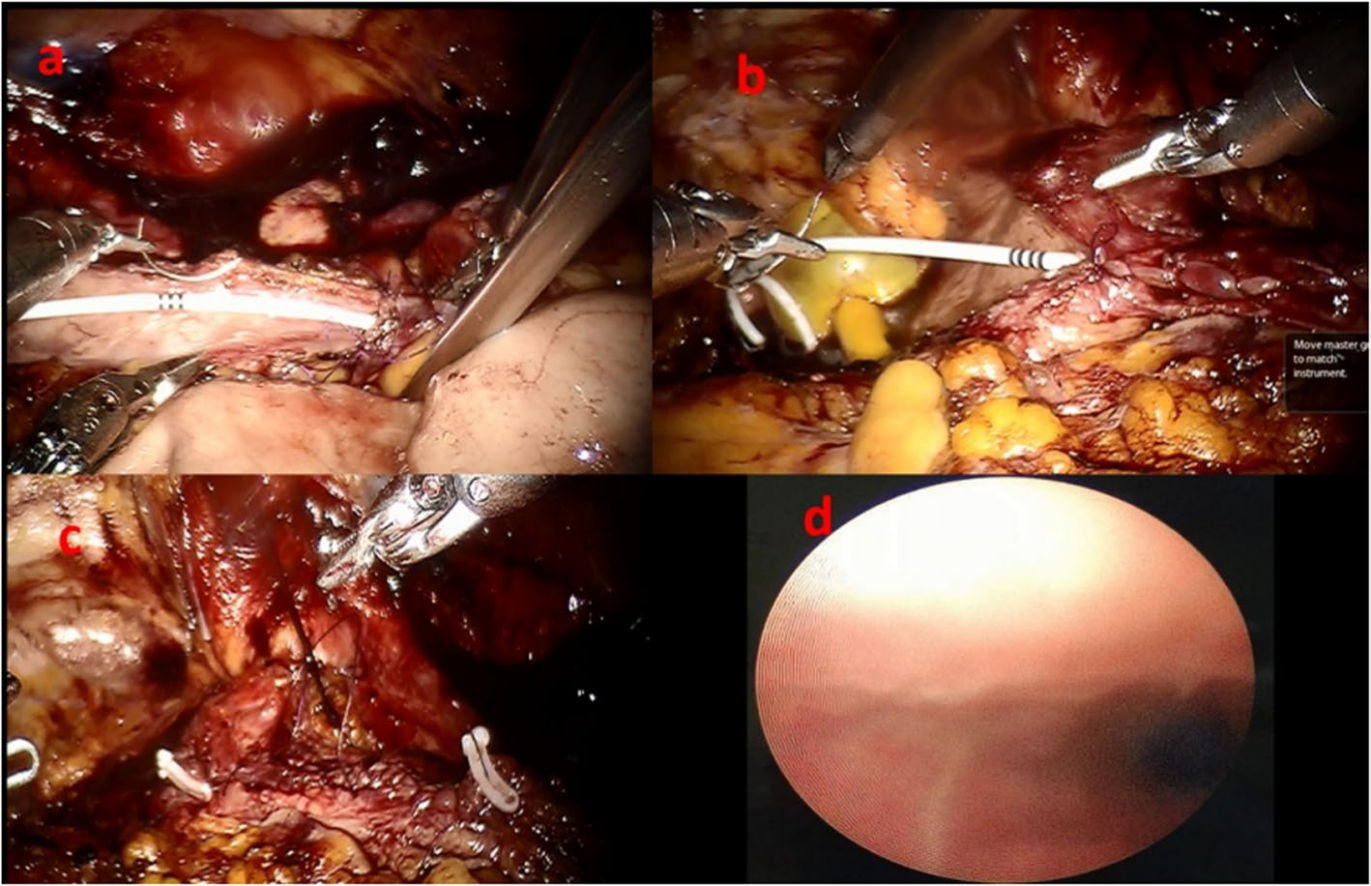
**a** Ureterovesical (flap) anastomosis. **b** Flap tubularized over a stent. **c** Completed flap. **d** Normal upper ureter through the flap at 1 year

Patient position was changed to low lithotomy position with steep Trendelenberg tilt. Five ports were placed as used for pelvic surgery (2 × 12 mm, 3 × 8 mm) utilizing previous port sites if possible. Robot was docked between the legs. Ureter was dissected till the bladder. Bladder was dropped down and vesicotomy done along the probable line of flap, bladder mucosa incised around the orifice and distal ureterectomy completed. Orifice rent closed with 2-0 polyglactin suture. Standard pelvic lymphadenectomy was done. After the frozen section confirmed negative margin, right superior vesical artery-based bladder flap (Boari flap) was created and refluxing ureterovesical anastomosis was done over a 6-Fr stent (Figs. 3 and 4). 22-Fr urethral catheter and abdominal drain were placed. Robot was undocked and specimens were removed.

The operative duration was 370 min and console time was 280 min. Estimated blood loss was 250 ml. Oral feeds were started on day 1, drain removed on day 4, and discharged on day 5. Urethral catheter was removed on day 14 and stent removed after 6 weeks.

Histopathology report showed T1b renal clear cell carcinoma, grade 2 with free margins and T3a (subserosa involved), and high-grade ureteric transitional cell carcinoma with margins free. Five lymph nodes were identified and uninvolved.

His nadir creatinine was 3.3 mg% at 1 year follow-up. MRI and cystoscopy with ureteroscopy at 1 year showed no evidence of recurrence.

## Discussion

Synchronous renal cell carcinoma and urothelial carcinoma is very uncommon [1]. Though many case reports of synchronous renal cell carcinoma and renal pelvic/calyceal urothelial cacinoma are available, synchronous renal and ureteric tumors are very rare [1, 5]. Most of the case reports are incidentally identified urothelial carcinoma lesions in the nephrectomy specimens of renal tumors. These patients are managed further with close follow-up of distal ureteric stump with wash cytologies and ureteroscopies or distal ureterectomy [5].

Partial nephrectomy is the standard for small renal tumors [2]. Though nephron sparing surgery is not usually offered for those with ureteric tumors, many recent studies indicate comparative recurrence rate and survival between patients undergoing segmental ureterectomy and nephroureterectomy [3, 6]. Low-grade and/or low-stage ureteric urothelial tumors, or those with risk of severe renal dysfunction post radical nephroureterctomy are preferred for nephron sparing surgery [6].

The main concern with ureteric urothelial tumors undergoing nephron sparing approach is recurrence of tumor. It is proven that recurrence after nephro ureterectomy for tumors in the kidney and upper ureter occurs in the distal ureter remnant—if not excised with bladder cuff. Even in those undergoing segmental ureterectomy, recurrence in mainly distal. It is very rare to have a recurrence proximal to the tumor site [6]. Hence, distal ureterectomy is an oncologically safe option for management of distal ureteric tumors.

The standard approach for ipsilateral renal and distal ureteric tumors would have been nephroureterectomy, if the renal function is normal. This individual had a low baseline GFR. The baseline GFR of 16 ml/min, indicates that he has a likelihood of lesser life expectancy (CKD stage 4) than those with higher GFR [7]. But with the addition of 12–14 ml GFR from the opposite kidney, he almost reaches 30 ml/min–grade 3 renal dysfunction from grade 4. Without the right kidney, he might deteriorate to grade 5 renal dysfunction sooner. The morbidity of dialysis dependence (with HBV sero positivity) has to be balanced against the chances of recurrence. Since the literature states that the incidence of recurrence does not depend on the radicality of the surgery, preserving renal function is of primary importance.

The prognosis is poor irrespective of the treatment approach in dual malignancies [1]. Follow-up of these individuals has to be strictly adhered to. Surveillance cystoscopy of the bladder and ipsilateral ureter (made fairly easy by Boari flap) every 3 months for 2 years in absolutely necessary. Later, the frequency may be reduced.

Minimally invasive approach, including robot-assisted procedures, is preferred, if necessary expertise is available for nephro sparing surgery, in renal tumors and in many instances for ureteric tumors [2, 3]. Partial nephrectomy with distal ureterectomy by open approach would need a long midline incision or two incisions. Minimally invasive approach considerably reduces the morbidity associated with the incision. Robot-assisted approach reduces the tissue trauma, aiding early recovery and better patient satisfaction.

This is the first case report of robot-assisted ipsilateral partial nephrectomy with distal ureterectomy in single stage.

## Conclusions

Robot-assisted partial nephrectomy with ipsilateral distal ureterectomy is feasible and a reasonable option with good oncological control, especially in those with renal dysfunction with synchronous renal and ureteric tumors.

## Data Availability

The data for current study is available from corresponding author at reasonable request.

## Abbreviations

GFR: Glomerular filtration rate
MRI: Magnetic resonance imaging
T2W: T2 weighted
CKD: Chronic kidney disease
HBV: Hepatitis B virus
